# Rapid detection of *Impatiens necrotic spot virus* from thrips vectors using reverse transcription-recombinase polymerase amplification

**DOI:** 10.1038/s41598-024-73078-4

**Published:** 2024-09-20

**Authors:** Shulu Zhang, Laura L. Hladky, Daniel K. Hasegawa

**Affiliations:** USDA – Agricultural Research Service, 1636 East Alisal Street, Salinas, CA 93905 USA

**Keywords:** Western flower thrips, *Impatiens necrotic spot virus*, Lettuce, Rapid detection, Reverse transcription-recombinase polymerase amplification (RT-RPA), Crude extract, Plant sciences, Molecular biology, Viral vectors

## Abstract

The plant virus, *Impatiens necrotic spot virus* (INSV), is an economically important pathogen of vegetables, fruits, and ornamental crops. INSV is vectored by the western flower thrips, *Frankliniella occidentalis*, a small insect pest that is globally distributed. In recent years, INSV outbreaks have reached epidemic levels in the Salinas Valley of California—an agriculturally rich region where most of the lettuce (*Lactuca sativa*) is produced in the United States. Due to the obligate nature in which virus transmission occurs, new tools that could rapidly detect INSV from thrips vectors would enhance our ability to predict where virus outbreaks may occur. Here, we report on the development of a reverse transcription-recombinase polymerase amplification (RT-RPA) assay that can detect INSV from individual thrips. The assay uses crude extraction methods, is performed at a single temperature of 42 °C, can be completed in 25 min, and provides sensitivity levels that are comparable to other available detection methods. When the assay was used on field populations of thrips, INSV was successfully identified and quantified from individual larvae and adults. The work provides a new cost-effective surveillance tool that can rapidly detect INSV from its insect vector and from plants.

**Supplementary Information:**

The online version contains supplementary material available at 10.1038/s41598-024-73078-4.

## Introduction

**Fig. 1:**
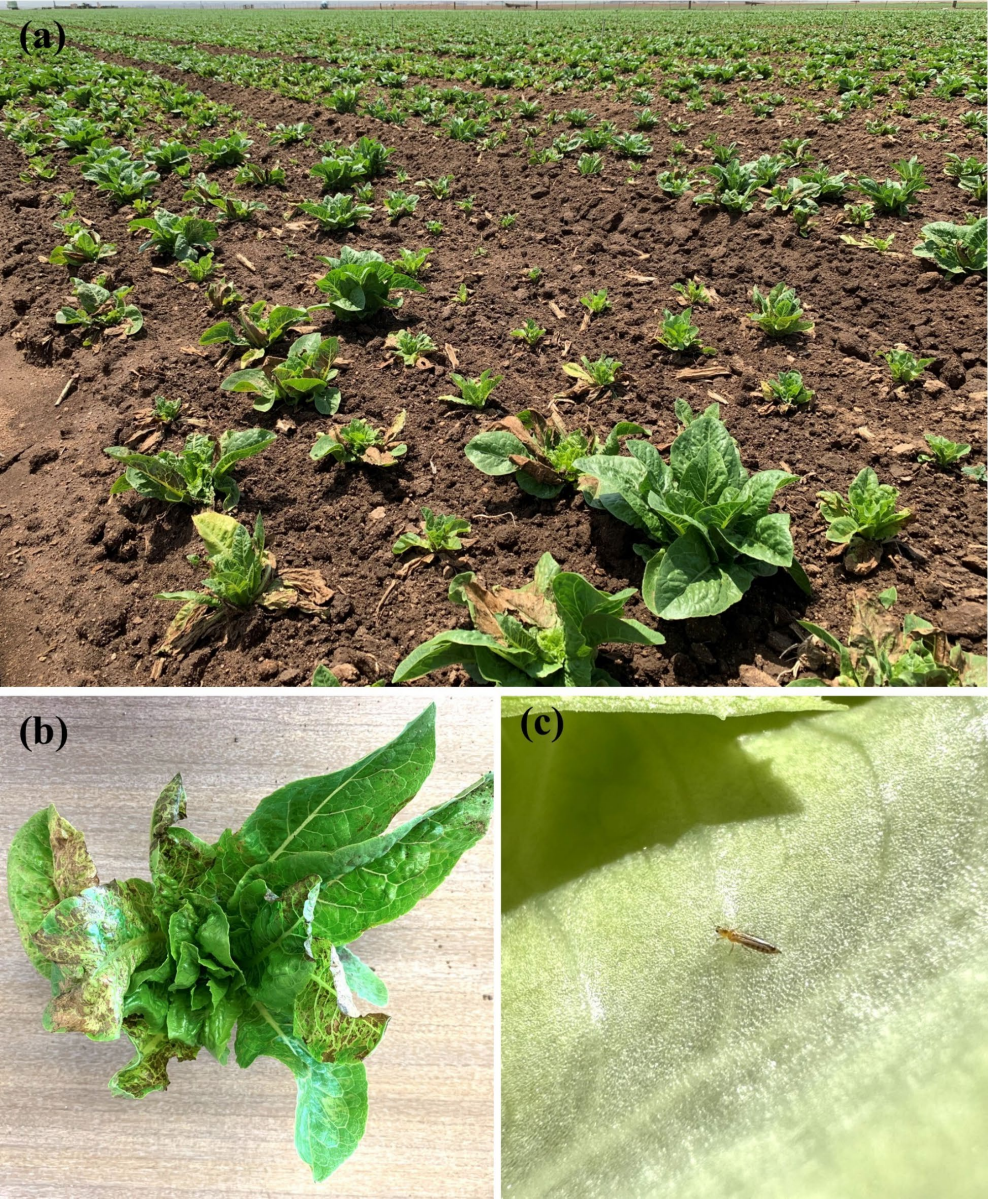
INSV-infected lettuce plants and associated western flower thrips. INSV-infected (**a**) field of romaine lettuce and (**b**) individual plant exhibiting typical symptoms of leaf necrosis, twisting of leaves, and stunted growth; (**c**) adult western flower thrips on a lettuce leaf.

Lettuce (*Lactuca sativa*) is a high-value leafy green vegetable that is worth over one billion U.S. dollars annually in the Salinas Valley of California, a region often referred to as the “Salad Bowl of the World”^1^. However, in recent years, lettuce production has incurred severe economic losses due to the re-emergence of a highly destructive plant virus, *Impatiens necrotic spot virus* (INSV), which is transmitted in a persistent circulative manner by a tiny insect vector^2,3^. While virus-resistant lettuce cultivars remain largely unavailable, effective management options for the insect vector and INSV-incited disease in lettuce are lacking. INSV, also known as *Orthotospovirus impatiensnecromaculae* in the family *Tospoviridae*, is a single-stranded RNA virus with a tripartite genome that includes the S (small), M (medium), and L (large) RNAs^4,5^. The S RNA is in an ambisense orientation and codes for the structural nucleocapsid protein N and non-structural protein S (NSs), a suppressor of gene silencing^6^. The M RNA is also ambisense and codes for the precursor of two structural glycoproteins (Gn and Gc) and non-structural protein M (NSm) that is involved in cell-to-cell movement in plants^7^. The L RNA is of antisense polarity and codes for the RNA-dependent RNA polymerase. Like other orthotospoviruses such as *Tomato spotted wilt virus* (TSWV, *Orthotospovirus tomatomaculae*), INSV replicates inside its insect vector and host plant, and can infect a wide range of plant species including weeds, fruits, vegetables, and ornamental crops; however, disease symptoms can vary depending on the host species and cultivars^2,8–17^. In lettuce, plants often exhibit necrotic leaf spots, stunted growth, and in some cases, plant death (Fig. 1). In addition to lettuce, INSV infection has impacted the production and marketability of numerous other important crops, including impatiens, begonia, tomato, potato, peanut, and blackberry^9,11–14^.

Western flower thrips (WFT), *Frankliniella occidentalis* (Pergande) (*Frankliniella*, *Thripidae*), is native to the southwestern United States but has since been established globally^18^. WFT is a small (1–2 mm), highly polyphagous pest that can infest a broad range of plant species, often leading to feeding damage, contamination issues, and transmission of viruses that impact the market quality of the commodity^18–20^. WFT exhibits a rapid life cycle of six developmental stages: egg, first and second larval instars, pre- and pro-pupae, and adult^21,22^. Critically, INSV must be acquired as larvae for virus transmission to occur during the active adult stage^21–24^. Due to its short reproductive cycle, high fecundity, large host range, and high virus transmission efficiency, management of INSV by targeting the vector has been insufficient as a standalone tactic^18–20,25^.

While detection of INSV from infected plants is an important component of disease diagnostics and management^20,25^, new tools that can rapidly detect INSV from both the vectoring insects and host plants would enhance our ability to predict the time and location of INSV outbreaks and respond accordingly with targeted management tactics. One approach is to identify vector populations that are associated with INSV, which could, in turn, provide insight as to when and where disease outbreaks will arise. Several molecular and serological detection methods^25–28^ and test kits (Agdia Inc., Elkhart, Indiana; BIOREBA AG, Switzerland) are available for the detection of INSV in plants; however, there is little information on the detection of INSV from its insect vectors.

Recombinase polymerase amplification (RPA) was first reported by Piepenburg et al. in 2006^29^ and has been shown to be a powerful tool for the rapid detection of specific DNAs or RNAs from plants, animals, humans, fungi, bacteria, and viruses^30–37^. RPA utilizes recombinase-assisted homologous pairing of primers and probes to their target DNA or RNA, and therefore, is highly specific^38^. The entire process can be rapidly performed at a relatively low and accessible temperature around 37 –44 °C. The availability of RPA-based commercial test kits (Agdia) and numerous research reports in recent years^34–36^ have also demonstrated qualitative and quantitative detections of pathogens in plants both in laboratories and from the field. However, there is little information on the use of RPA for the detection of viruses from insects. Thus, detection of INSV from its insect vector and host plants using isothermal RPA can provide a new rapid and accurate diagnostic tool for monitoring the presence of INSV in fields and enhance our ability to mitigate the disease effectively.

In this study, we utilized the isothermal RT-RPA technology and developed a new molecular diagnostic assay for rapid and cost-effective detection of INSV in WFT vectors using crude extraction methods. We also demonstrate that the assay is capable of quantitatively detecting the presence of INSV from single thrips collected from the field. To our knowledge, this is the first application of RT-RPA for the detection of INSV from its insect vector and host plants.

## Results

### Design, selection, and specificity of RT-RPA primers and probes

Genomic sequences of all three INSV RNA segments were analyzed using the BLAST program^39^ for unique conservative regions across INSV strains and isolates. From one of the nucleotide sequence-conserved regions in the N gene of the S RNA, several forward and reverse primers and internal fluorogenic probes were designed. The primers and probes were screened for their ability and specificity to detect INSV using in vitro transcribed RNA and purified total RNAs, as well as crude extracts from INSV-infected thrips and lettuce. The primers INSV RPA-F5 and INSV RPA-R2-2, and the probe INSV RPA exo-P1 (Supplementary Information—Table S1) were selected for their specific and strong performance in the detection of INSV. The two RT-RPA primers amplify a region of the N gene of the INSV S RNA at nucleotide positions 2591–2758 (NCBI NC_003624), producing a 168 bp amplicon. BLAST analysis revealed that the RT-RPA primers and probe are specific only to INSV (Supplementary Information—Fig. S1 and Table S2) and do not align to other organisms.

### Optimization of reaction conditions

The INSV primers, RPA-F5 and R2-2 and probe, exo-P1 were optimized to a working concentration of 240 nM, 240 nM, and 56 nM, respectively, and were subsequently used throughout the study. Various reaction conditions, including reaction temperature (39 °C and 42 °C), time (20 min and 25 min), and volume (20 µl, 25 µl, and 50 µl) were tested. As a result, the RT-RPA assay was finalized and performed at a constant temperature of 42 °C for 20 min using the AmpliFire^®^ Isothermal Fluorimeter (Agdia). Both 25 µl and 50 µl volumes produced similar results and the 25 µl reaction volume was adopted for all reactions except for the thrips and plant samples collected from the field, as well as the standard curve, which were tested in 50 µl reaction volumes. At the time of the study, lyophilized pre-mixed reactions for performing RPA were only available in 50 µl volumes, and therefore, were chosen for its convenience when testing the thrips and lettuce samples collected from the field.

### Crude extractions from thrips and lettuce

**Fig. 2:**
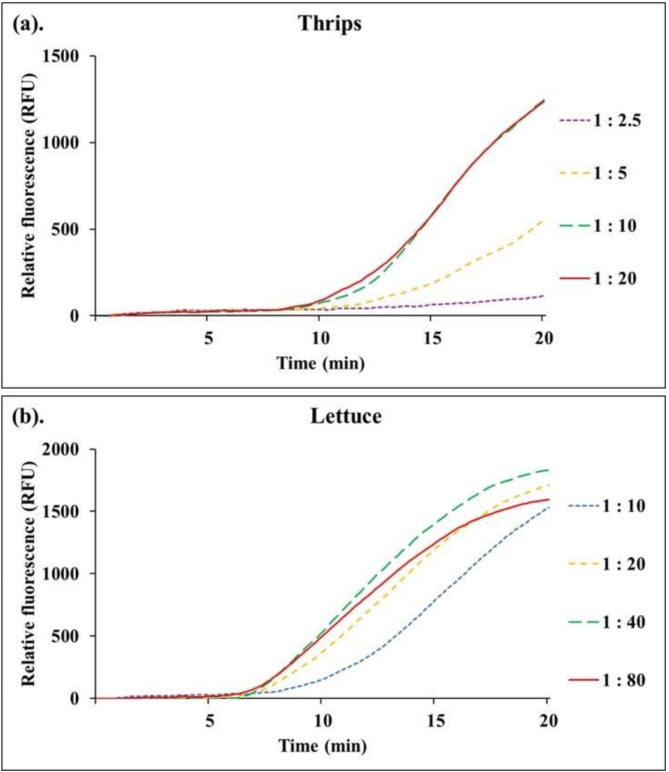
Optimization of thrips and lettuce crude extracts for RT-RPA. (**a**) Crude extracts from 10 healthy thrips were diluted in different volumes of TEB1 buffer: 25 µl (1:2.5 thrips to buffer volume ratio), 50 µl (1:5), 100 µl (1:10), and 200 µl (1:20) and then spiked with 100 fg of in vitro transcribed INSV RNA and tested using RT-RPA. (**b**) Crude extracts from 300 mg of INSV-infected tissue were diluted at a ratio of 1:10 (300 mg of tissue to 3 ml), 1:20, 1:40, and 1:80 and tested using RT-RPA.

**Fig. 3:**
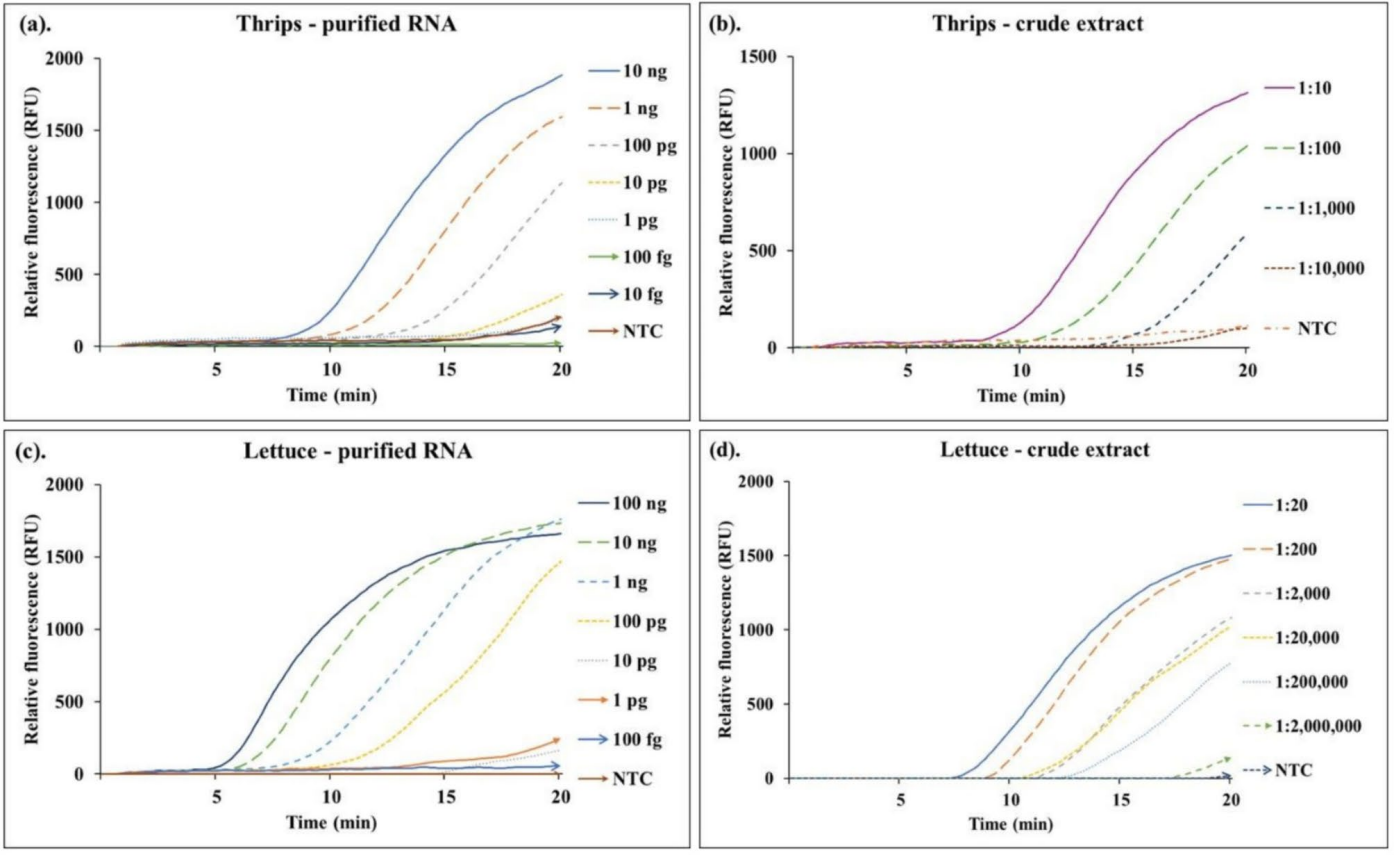
Sensitivity assay for RT-RPA in thrips and lettuce using crude extracts and purified RNA. (**a**) Thrips purified total RNA was diluted six times from 10 ng to 10 fg; (**b**) Thrips crude extracts were diluted three times, each at a 10-fold dilution from a single thrips; (**c**) Lettuce purified total RNA was diluted six times from 100 ng to 100 fg; (**d**) Lettuce crude extracts were diluted five times, each at a 10-fold dilution. NTC = no template control.

Crude nucleic acid extracts of thrips and lettuce were tested using several methods. The extraction buffers that were tested included RLC buffer (Qiagen), 1x PBS (Phosphate Buffered Saline, pH 7.4), and TEB1 (Thrips Extraction Buffer 1). For thrips, TEB1 buffer produced the strongest fluorescent signal, followed by 1x PBS, while the RLC buffer completely inhibited the fluorescent signal and reaction. A comparison of TEB1 buffer concentrations at 1x and 0.2x determined that the 1x concentration yielded the strongest fluorescent signal (Supplementary Information—Fig. S2). Lastly, the fluorescent signal of the RT-RPA assay was optimal when 10 thrips were extracted using 100–200 µl of TEB1 buffer (1:10 or 1:20 extraction ratio) (Fig. 2a). Thus, we adopted a protocol that uses 20 µl of TEB1 buffer when a single thrips is extracted. For samples that included more than one thrips, 10 µl of TEB1 buffer was added for every thrips. For lettuce crude extractions, dilutions of 1:20 (mg tissue to ml TEB1 buffer) and 1:40 exhibited similar fluorescent signal onset times and both achieved a high RFU value, suggesting a lack of inhibition in the samples (Fig. 2b). Therefore, we selected the lower dilution ratio (1:20) because of its advantages when testing samples with lower starting virus titers. In addition, INSV was detected when either in vitro transcribed INSV RNA (Supplementary Information—Fig. S3) or total RNAs (Fig. 3) from thrips and lettuce were used as reaction templates.

### Specificity and sensitivity assays

**Fig. 4:**
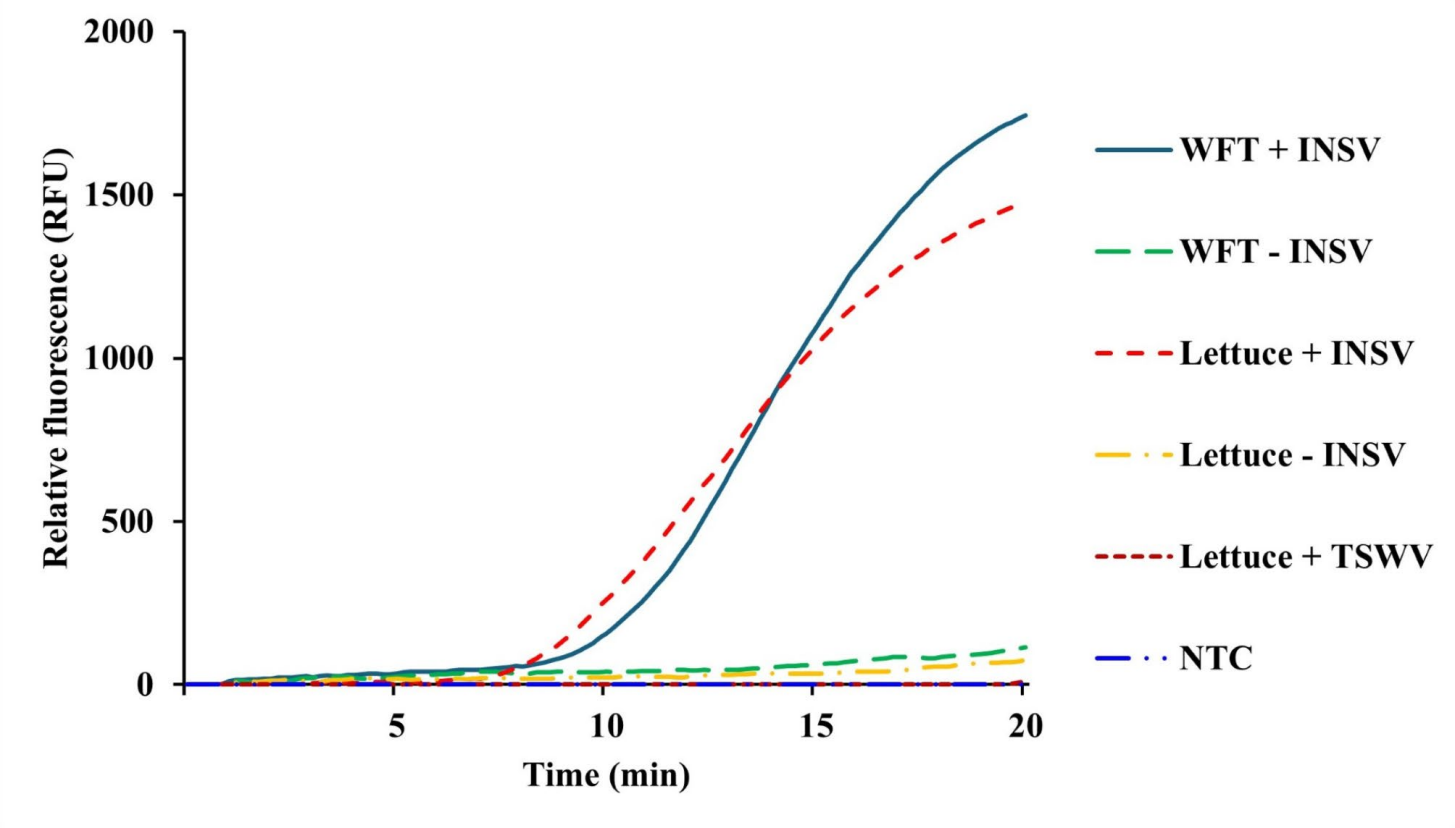
Specific detection of INSV from infected thrips and lettuce. The samples for this assay are as follow: WFT + INSV = INSV-infected western flower thrips; WFT - INSV = uninfected western flower thrips; Lettuce + INSV = INSV infected lettuce; Lettuce - INSV = uninfected lettuce; Lettuce + TSWV = TSWV-infected lettuce; NTC = no template control.

To validate the specificity of the assay, single healthy thrips and lettuce plants, as well as lettuce plants infected with TSWV—an orthotospovirus closely related to INSV, were tested using the RT-RPA protocol. For all samples, no cross reactions were observed, while thrips and lettuce samples that were infected with INSV produced the expected amplification signal (Fig. 4).

To determine the sensitivity of the assay, in vitro transcribed INSV RNA, purified total RNAs, and crude extracts from thrips and lettuce were used as reaction templates for the RT-RPA protocol. For the in vitro transcribed RNA, INSV was detected from as little as 1.0 femtogram (fg) (Table 1 and Supplementary Information—Fig. S3). From the purified total RNAs of thrips and lettuce, the virus was detected from as little as 10 picograms (pg) and 100 pg of the total RNA, respectively (Fig. 3a and c, and Table 1). When crude extracts were used as the reaction template, INSV was detected from a single thrips when the extract was diluted at a ratio between 1:10 to 1:1,000 (Fig. 3b; Table 1). Similarly, the assay was able to detect INSV from crude extracts of lettuce that were diluted at a ratio between 1:20 and 1:200,000 (Fig. 3d; Table 1). The results demonstrate specific and sensitive detection of INSV from several template sources and provide guidelines for preparing thrips and lettuce samples for RT-RPA protocols.

**Table 1:** Detection of INSV from a variety of template sources via the RT-RPA assay.

Thrips: purified RNA		Thrips: crude extract		Lettuce: purified RNA		Lettuce: crude extract		RNA transcript	
Amount	Result	Extraction ratio	Result	Amount	Result	Extraction ratio	Result	Amount	Result
10 ng	+	1:10	+	100 ng	+	1:20	+	100 pg	+
1 ng	+	1:100	+	10 ng	+	1:200	+	10 pg	+
100 pg	+	1:1,000	+	1 ng	+	1:2,000	+	1 pg	+
10 pg	+	1:10,000	−	100 pg	+	1:20,000	+	100 fg	+
1 pg	−	NTC	−	10 pg	−	1:200,000	+	10 fg	+
100 fg	−			1 pg	−	1:2,000,000	−	1 fg	+
10 fg	−			100 fg	−	NTC	−	100 ag	−
NTC	−			NTC	−			NTC	−

Rapid detection of *Impatiens necrotic spot virus* from thrips vectors using reverse transcription-recombinase polymerase amplification.

### Comparison of RT-RPA with other detection methods

To compare the sensitivity of the RT-RPA assay to other existing detection methods, reactions were set up in parallel with RT-PCR, RT-qPCR, DAS-ELISA and ImmunoStrip protocols. A crude extract from 14 INSV-infected thrips was prepared, serially diluted from 1:10 to 1:100,000, and used as the reaction template for all the methods except RT-PCR. Both RT-RPA and RT-qPCR detected INSV from crude extracts that were diluted at 1:1,000, while DAS-ELISA and ImmunoStrips were only able to detect INSV when diluted 1:100 and 1:10, respectively (Table 2). For RT-PCR, using crude extracts from thrips appeared to show some inhibition, thus, total RNA was purified from the serially diluted crude extracts and then used as the reaction template. Despite the additional purification steps, INSV was only detected at a dilution ratio of 1:10 using RT-PCR (Table 2). These results indicated that the RT-RPA assay was equally as sensitive to the RT-qPCR method and provided greater sensitivity than RT-PCR and both serological detection methods.

**Table 2:** Detection of INSV from thrips using different methods.

Sample #	Dilution ratio	RT-RPA	RT-qPCR	RT-PCR	ELISA	ImmunoStrip
1	1:10	+	+	+	+	+
2	1:100	+	+	−	+	−
3	1:1,000	+	+	−	−	−
4	1:10,000	−	−	−	−	−
5	1:100,000	−	−	−	−	−
6	NTC	−	−	−	−	−

Rapid detection of *Impatiens necrotic spot virus* from thrips vectors using reverse transcription-recombinase polymerase amplification.

### Field implementation of the RT-RPA assay for detecting INSV from thrips and lettuce

To establish protocols for the quantification of INSV from field-sampled thrips and lettuce, in vitro transcribed RNA was serially diluted from 10 nanograms (ng) to 100 attograms (ag) and assayed. A minimum of 1.0 fg of the in vitro transcribed target RNA was detected (Supplementary Information—Fig. S3) and a standard curve was generated with the formula of y = 0.10x + 0.57 (R^2^ = 0.99) for calculating the number of targeting INSV RNA molecules from field-collected thrips (Supplementary Information—Fig. S4).

In September 2023, 15 lettuce plants exhibiting INSV symptoms were collected from a commercial field in the Salinas Valley of California, all of which tested positive for INSV by RT-RPA and DAS-ELISA. Furthermore, 26 thrips of different developmental stages (4 larvae, 7 pupae, and 15 adults) were simultaneously collected from the same plants and processed individually in TEB1 buffer. The crude extracts were used in the RT-RPA assay and INSV was successfully detected from 4/4 larvae, 7/7 pupae, and 10/15 adults. The average copy numbers of the target INSV RNA molecules were highest in thrips larvae (3.4 × 10^7^), followed by pupae (4.3 × 10^6^), and then adults (5.3 × 10^5^) (Table 3). In addition, more than 71 adult thrips were also collected in groups consisting of 3–12 adults from the same lettuce plants and tested using RT-RPA and RT-qPCR. All groups tested positive for INSV using both methods and copy numbers of the target INSV RNA molecules ranged from 1.1 × 10^5^ to 2.1 × 10^7^ with an average of 4.7 × 10^6^ copies per batch of thrips (Data not shown).

**Table 3:** Detection and copy numbers of INSV in single field thrips using RT-RPA.

Developmental stage	# Of thrips	Average onset time (min) ± Standard error	Copy # per thrips
Larvae	4	7.9 ± 1.2	3.4 × 10^7^
Pupae	7	9.7 ± 1.8	4.3 × 10^6^
Adult	10	12.1 ± 2.8	5.3 × 10^5^

## Discussion

Western flower thrips is a tiny polyphagous pest that inflicts damage to agricultural and horticultural crops through direct feeding and the transmission of plant pathogenic viruses. In recent years, INSV has become an important pathogen of lettuce grown in the U.S. and Europe^3,40^ and naturally infects a wide range of plant species; however, disease symptoms are not always clear^2,9,17,40,41^. Thus, symptom-based diagnostics is not always reliable, further challenging the implementation of effective mitigation and prevention strategies. One strategy that could enhance our ability to identify risks for virus outbreaks in lettuce and other crops is by screening the vector to identify emerging thrips populations that are associated with INSV. Tools that rapidly detect viruses from their insect vectors could facilitate our ability to respond with appropriate management measures across time and space. The reverse transcription-recombinase polymerase amplification (RT-RPA) assay developed in the current study provides a new tool that is fast, sensitive, and cost-effective for identifying INSV from thrips vectors and host plants. The work has practical implications for improving the surveillance of insects and insect-transmitted viruses.

RPA was first reported by Piepenburg et al. in 2006^29^ and has since become increasingly utilized for scientific studies and industrial applications^32,33,35,36,42^. RPA possesses several useful features including, (1) the use of recombinase-assisted, homology-based pairing of primers and probes, (2) a single relatively low and accessible temperature at which amplification occurs, (3) rapid amplification that occurs in minutes, and (4) high tolerance to endogenous inhibitors, allowing for the use of crude extracts as reaction templates instead of DNA or RNA purified with traditional purification methods that are more expensive and time-consuming^30,34,43^. Thus, the RPA method has become a leading diagnostic tool for the identification of pathogens in humans, animals, and plants^32^. However, there is little information available on the use of RPA technologies in insects. The current study describes the development and application of a new RT-RPA assay for the rapid detection of INSV from its insect vector and host lettuce plants. To our knowledge, this is the first report on the detection of INSV using RT-RPA from insects and plants.

The RT-RPA assay provides qualitative and quantitative data for virus identification and was performed using a simple fluorescent reader. The simplicity and speed of the assay also facilitates its broad and rapid application^36,44^. The RT-RPA primers and probes were designed from a region of the N gene of INSV S RNA that is highly conserved across INSV strains and isolates from across the world. The specific detection of INSV was revealed from in-silico sequence analysis and then demonstrated experimentally, while the sensitivity was determined by using several types of template matrices including the crude extracts and pure total RNA of thrips and lettuce plants. The assay was able to detect a minimum of 1.0 fg of the in vitro transcribed INSV RNA, which has been achieved from only a few other reported RT-RPA assays^30,45^. Using the crude extract and total RNA from thrips, the sensitivity of the RT-RPA assay was greater than conventional molecular detection (RT-PCR) and serological detection (DAS-ELISA and ImmunoStrip) methods and was comparable to RT-qPCR methods. The ability to detect INSV at such a specific and sensitive level compared to other methods provides an additional diagnostic tool for accurately identifying INSV from thrips and plants. The RT-RPA can also be used for quantitative applications, however, only a few reports have described its utility^36,44,45^. Here, the RT-RPA assay was used to quantitate INSV from individual thrips larvae, pupae, and adults, which could be applied to future studies to understand vector-virus relationships.

Considering the high population densities that western flower thrips can achieve in the environment, we aimed to minimize the cost of the assay to permit its use as a diagnostic tool for screening large samples of thrips. Protocols were developed to use crude extracts, which eliminated the requirement for costly nucleic acid purification kits. Using this approach, the assay was sensitive enough to detect INSV from a single thrips. Additionally, INSV was still detectable from a batch of thrips containing a single viruliferous thrips and 19 non-viruliferous thrips (data not shown). At the time of this study, the RT-RPA assay cost ~$2 per reaction, which includes the TwistAmp^®^ exo kit ($1.35) and RPA primers and probe ($0.22). Also, at the time of the study, the cost of other INSV diagnostic methods were ~$2.5 per RT-qPCR reaction (plus an additional $6 to purify the RNA using a commercial kit), $1 per ELISA well, and $7.5 per ImmunoStrip. In terms of time, both the RT-RPA and ImmunoStrip assays required less than 25 min to complete, while RT-PCR, RT-qPCR, and ELISA required several hours to 2 days to complete^36^. Thus, the newly developed RT-RPA assay provides a rapid and cost-effective tool for the identification of INSV from thrips and plants.

Recent outbreaks of INSV have occurred in places including the Salinas Valley of California and have resulted in significant crop losses^2,3,9,17,46^. Due to the large host range of the vector and virus, the creation of new diagnostics tools that identify thrips populations that are associated with INSV will enhance our ability to develop targeted management strategies to minimize disease outbreaks. Furthermore, the use of RT-RPA technologies to screen vector populations for plant pathogens could be especially useful for identifying invasive and quarantined pests to aid in their early detection and mitigation.

## Materials and methods

### Plant material and virus sources

Romaine lettuce (*Lactuca sativa*) var. Parris Island was maintained in greenhouses at 22 °C with natural lighting. Healthy plants were maintained in a thrips-proof cage in a single greenhouse, while INSV-infected lettuce was maintained in a separate greenhouse and was used as source materials for developing the assay. Isolates of *Impatiens necrotic spot virus* (INSV, also known as *Orthotospovirus impatiensnecromaculae*) originated from fields in the Salinas Valley, which were previously reported^3^. The *Tomato spotted wilt virus* (TSWV, also known as *Orthotospovirus tomatomaculae*) isolate was collected from the Salinas Valley from lettuce in 2023 and was confirmed using both TSWV-specific ImmunoStrip and ELISA assays (Agdia Inc., Elkhart, IN) in the laboratory. Fifteen symptomatic lettuce plants were collected from a commercial field in the Salinas Valley in 2023 and confirmed for the presence of INSV using DAS-ELISA (Agdia) and RT-RPA.

### Thrips maintenance and virus acquisition

A colony of western flower thrips (WFT) (*Frankliniella occidentalis*) was maintained in the laboratory on green beans at 22 ± 2 °C under 24 h light. For virus acquisition studies, first instar larvae were collected from green bean pods and transferred to a plastic cup containing INSV-infected lettuce leaves and incubated at 22 °C for 48 h. Thrips were transferred back to healthy green bean pods under the same conditions until adulthood. Viruliferous and non-viruliferous thrips from the primary colony were collected into 1.5-ml tubes using a paintbrush and either processed immediately for extraction or stored in 95% ethanol at -80 °C until use.

### Preparation of purified RNA and crude extracts from thrips and lettuce

Lettuce tissue and thrips were homogenized in a 1.5-ml microcentrifuge tube using sterile micro pestles. Total RNA was extracted using the RNeasy Plant Mini Kit (Qiagen, Germantown, MD) following manufacturer’s instructions. RNA was eluted in 50 µl and concentrations were determined using a Nanodrop 2000 Spectrophotometer (Thermo Fisher Scientific, Waltham, MA). To obtain crude extracts, more than 97 thrips and over 15 lettuce plants were homogenized as above in TEB1 (Thrips Extraction Buffer 1: 68.5 mM NaCl, 1.35 mM KCl, 5 mM Na_2_HPO_4_, and 0.9 mM KH_2_PO_4_, pH 7.4). A single thrips was extracted in 20 µl of TEB1 (1:20). When more than one thrips was extracted in a single tube, 10 µl of TEB1 was added for every thrips in the sample (1:10). Lettuce samples were homogenized in a mesh bag at a ratio of 1:20 (or 50 mg tissue to 1.0 ml of TEB1 in a microcentrifuge tube).

### In vitro RNA transcription, quantification, and generation of a standard curve.

A DNA fragment of 524 bp (NC_003624 nucleotides 2401–2924) from the INSV S RNA flanked with the T7 promoter sequence (5’-TAATACGACTCACTATAGGG-3’) was used as the template for in vitro RNA transcription using the MEGAscript^®^ RNAi Kit (Invitrogen, Carlsbad, CA) to produce single stranded RNA (ssRNA). Briefly, the ssRNA was transcribed for 4 h at 37 °C, and treated with DNase I for 30 min at 37 °C. The ssRNA transcripts were precipitated with lithium chloride and resuspended in nuclease-free water. The concentration of RNA was determined on a Nanodrop 2000 Spectrophotometer. Copy numbers of the transcribed RNA molecules were calculated using the formula: ssRNA copy number = [mass of ssRNA (g) / (number of ribonucleotides of ssRNA x 320.47 + 18.02)] x 6.022 × 10^23^. For sensitivity assays, the in vitro transcribed RNA was serially diluted 10-fold with water 8 times from 10 ng to 100 ag per µl and subjected to the RT-RPA assay. Fluorescence readings were plotted against time. The template RNA concentrations from tested samples were calculated using the formula Y = 0.10X + 0.57 from the standard curve and the copy numbers of the target INSV RNA molecules from thrips were determined as above.

### Serological assays for detecting INSV from thrips and lettuce

Lettuce tissues were extracted and tested for the presence of INSV using INSV-specific DAS-ELISA and ImmunoStrip tests following manufacturer′s instructions with modifications of sample extraction methods (Agdia). To prepare the samples, thrips were homogenized in TEB1 as previously described, and further diluted at 1:10 ratios with the included GEB1 buffer for DAS-ELISA or SEB1 buffer for the ImmunoStrip assay. For the DAS-ELISA, 10 µl of the crude extract prepared in TEB1 from 14 adult thrips was mixed with 90 µl of GEB1. Samples were tested in triplicate and absorption was measured at 405 nm using a BioTek ELX800 Microplate reader (BioTek Instruments Inc., Winooski, VT). A sample was considered positive for INSV when the absorption value was 2.5 times the value of the negative control (uninfected thrips or lettuce). Similarly, for the ImmunoStrip assay, 10 µl of the same TEB1 crude extract was mixed with 90 µl of SEB1.

### Thermal amplification assays for detecting INSV from thrips and lettuce

For RT-PCR, purified RNA was reverse-transcribed using the gene-specific INSV PCR reverse primer and M-MLV reverse transcriptase (Promega, Madison, WI) at 42 °C for one hour to produce cDNA. PCR was performed to amplify a 524 bp region of the nucleoprotein (N) gene of INSV S RNA. PCR reactions included 2.5 µl of cDNA, 200 nM forward primer INSV PCR-F and 200 nM reverse primer INSV PCR-R (Supplementary Information—Table S1)^17^, and 12.5 µl GoTaq Green Master Mix (Promega) in a final volume of 25 µl. Reaction conditions included an initial incubation at 95 °C for 2 min, followed by 35 cycles of 95 °C/30 seconds, 55 °C/30 seconds, and 72 °C/60 seconds in an Eppendorf MasterCycler Nexus Thermal Cycler (Eppendorf, Enfield, CT). PCR products were separated through agarose gel electrophoresis and visualized using SYBR Safe DNA Gel Stain (Thermo Fisher Scientific). RT-qPCR was performed using the iTaq Universal Probes One-Step Kit (Bio-Rad, Hercules, CA) following the manufacturer’s protocol. Briefly, 1 µl of total RNA from thrips was used in a 20 µl reaction that included 250 nM forward primer INSV qPCR-F, 250 nM reverse primer INSV qPCR-R, and 250 nM probe INSV qPCR-P1 (Supplementary Information—Table S1). The primers and probe for the RT-qPCR were designed, optimized, and validated in the laboratory for the detection of INSV (Supplementary Information—Table S3). The RT-qPCR reactions were performed at 50 °C for 10 min and 95 °C for 2 min, followed by 40 cycles of 95 °C for 10 s and 60 °C for 30 s on a CFX Opus 96 Real-Time PCR System (Bio-Rad).

### Development of the RT-RPA assay

All RT-RPA primers and probes were designed from a nucleotide sequence-conserved region of the N gene in the S RNA segment and synthesized by Integrated DNA Technologies, Inc. (IDT, San Diego, CA). The primers and probe selected and used throughout the remainder of the study were INSV RPA-F5, INSV RPA-R2-2, and INSV RPA exo-P1 (Supplementary Information—Table S1 and Fig. S1). All reactions were performed using the TwistAmp^®^ exo kits (liquid and solid) (TwistDX Ltd, Maidenhead, UK) following manufacturer’s protocols. The exo liquid kit provides options for users to perform RPA reactions in desirable volumes such as 25 µl, which costs less than the 50 µl reactions commercially available as lyophilized reaction pellets in the exo solid kit. RT-RPA reactions included 240 nM INSV RPA-F5 primer, 240 nM INSV RPA-R2-2 primer, 56 nM for INSV RPA exo P1 probe, 200 µM each of dNTPs (Promega) (except for the lyophilized reaction pellets, which include pre-added dNTPs), and 4 U/µl of M-MLV Reverse Transcriptase (Promega). One microliter of purified RNA or crude extracts per 25 µl of a reaction was added to the reaction mix before adding the reaction activation solution. The reaction was incubated at 42 °C for 20 min using the AmpliFire^®^ isothermal fluorometer following the company’s instructions (Agdia).

### RT-RPA sensitivity assay

Purified total RNAs and crude nucleic acid extracts from thrips and lettuce, as well as in vitro transcribed INSV RNA were used as templates for the RT-RPA assay. All templates were serially diluted 10-fold, 6 times from 10 ng to 10 fg for the thrips purified RNA, 3 times from 1:10 to 1:10,000 for the thrips crude extract, 6 times from 100 ng to 100 fg for the lettuce purified RNA, and 5 times from 1:20 to 1:2,000,000 for the lettuce crude extract. One microliter of each dilution was used for the assay. TEB1 was used as the no-template-control (NTC).

### Comparison of RT-RPA to other detection methods

The RT-RPA assay was compared to several available detection methods including RT-PCR, RT-qPCR, DAS-ELISA and ImmunoStrip. A common crude nucleic acid extract was prepared by grinding 14 thrips in 140 µl of TEB1, serially diluted 10-fold, 4 times from 1:10 to 1:100,000 and used as the reaction template for the comparison with RT-qPCR, ELISA and ImmunoStrip, while total RNA purified from these dilutions was used as the reaction template for RT-PCR. For the RT-RPA and RT-qPCR assays, crude extracts were serially diluted in the same extraction buffer (TEB1) six times and 1 µl from each dilution was used as the reaction template. For DAS-ELISA, 10 µl of the same initial crude extract in TEB1 was mixed with 90 µl of the DAS-ELISA sample extraction buffer GEB1, followed by serially diluting in the GEB1 six times. Each dilution with a total of 100 µl per well on the 96-well microplate was used for the DAS-ELISA assay (Agdia). For ImmunoStrip testing, 10 µl of the initial crude extract in TEB1 was mixed with 90 µl of the ImmunoStrip extraction buffer SEB1, followed by serially diluting 10-fold, six times in SEB1. A total of 100 µl was used for the ImmunoStrip assay (Agdia). For RT-PCR, 10 µl of the original crude extract in TEB1 was serially diluted 10-fold, six times in TEB1 to a final volume of 100 µl. Total RNA was then purified from each dilution and eluted in 30 µl of elution buffer (Qiagen). 1.5 µl of total RNA was applied to the 2-step RT-PCR reaction and 2.5 µl of cDNA was used for PCR as described above^17^.

### Testing of field-collected thrips and lettuce

Romaine lettuce plants exhibiting INSV symptoms were collected from commercial fields in the Salinas Valley in September 2023 and brought back to the laboratory. Leaf tissues were extracted and tested for the presence of INSV using ELISA and the RT-RPA assay as described above. From the same lettuce plants, a total of 26 thrips: 4 larvae, 7 pupae, and 15 adults were individually collected, separated, and transferred to 95% ethanol in a 1.5 ml microcentrifuge tube using a paintbrush. For extraction, ethanol was removed, and thrips were individually homogenized in TEB1 and assayed. Additionally, more than 71 of adult thrips were collected in groups containing 3–12 thrips per group and extracted in the same manner before testing using RT-RPA and RT-qPCR. Copy numbers of target INSV RNA molecules from single and grouped thrips were calculated using the standard curve (Supplementary Information—Fig. S4) and its derivative formula.

## Supplementary Information

Below is the link to the electronic supplementary material.

Supplementary Material 1

## Data Availability

Supplementary Figures and Tables are provided with this article. Further inquiries should be sent to the corresponding author D.K.H.
