# Supplementary material for: Rapid detection of Impatiens necrotic spot virus from thrips vectors using reverse transcription-recombinase polymerase amplification
Source: Sci Rep. 2024 Sep 20;14:21946. doi: 10.1038/s41598-024-73078-4 (PMC11415493; doi:10.1038/s41598-024-73078-4)
Supplement: Supplementary file 1 — Supplementary Material 1 [file 41598_2024_73078_MOESM1_ESM.pdf]

Supplementary Information

Rapid detection of *Impatiens necrotic spot virus* from thrips vectors using reverse transcription-recombinase polymerase amplification

Shulu Zhang, Laura L. Hladky, and Daniel K. Hasegawa*

USDA – Agricultural Research Service, 1636 East Alisal Street, Salinas, California 93905.

*Corresponding author: Daniel K. Hasegawa, Email: daniel.hasegawa@usda.gov

Legends

Figure S1. Sequence alignments of the target region of the INSV S RNA from 24 representative strains and isolates (also see Table S2). The INSV isolates were chosen based on their availability of complete S RNA nucleotide sequences in the NCBI database. The nucleotide numbers 1-168 (1-84, top; 85-168, bottom) correspond to the nucleotide numbers 2,591 to 2,758 from NCBI database NC_003624. Differences in nucleotides are highlighted in red and the locations of the primers (RPA-F5 and RPA R2-2) and probe (RPA exo-P1) used for the RT-RPA assay are indicated by arrows.

Figure S2. Detection of INSV from crude extracts of thrips prepared in various extraction buffers using RT-RPA. RLC, broken blue line; 1x PBS, broken orange line; 1x TEB1, solid yellow line, 0.2x TEB1, broken grey line.

Figure S3. Detection of INSV from *in vitro* transcribed RNA using the RT-RPA assay. The RNA was serially diluted from 10 ng to 100 ag. RT-RPA amplification onset times were used for producing the standard curve (see Fig. S4). NTC, no template control.

Figure S4. Standard curve of RT-RPA from serial dilutions of the *in vitro* transcribed RNA. Amounts of the RNA per reaction are given on the right side of Fig. S3. The linear relationship was established using logarithms of the template concentrations (ng) against their amplification onset times (min).

Table S1. List of the primers and probes used for RT-RPA, RT-qPCR, and RT-PCR assays in the study. The names, nucleotide (Nt) sequences, and locations on the INSV S RNA segment (NCBI Reference Sequence: NC_003624.1) are given in the table.

Table S2. List of 24 INSV strains and isolates used for the sequence alignments in this study. The NCBI database accession numbers (Sequence ID), virus strain/isolate names, countries of virus origin, and host plant species are provided.

Table S3. Detection of INSV from plant samples by RT-qPCR and DAS-ELISA. For RT-qPCR, a Cq cutoff value of 35 was used, while for DAS-ELISA, a sample was considered positive if the absorbance reading was 2.5 times of the negative controls. The results from the RT-qPCR were fully validated by the commercial DAS-ELISA test (Agdia).

Figure S1. Sequence alignments of the target region of the INSV S RNA from 24 representative strains and isolates.

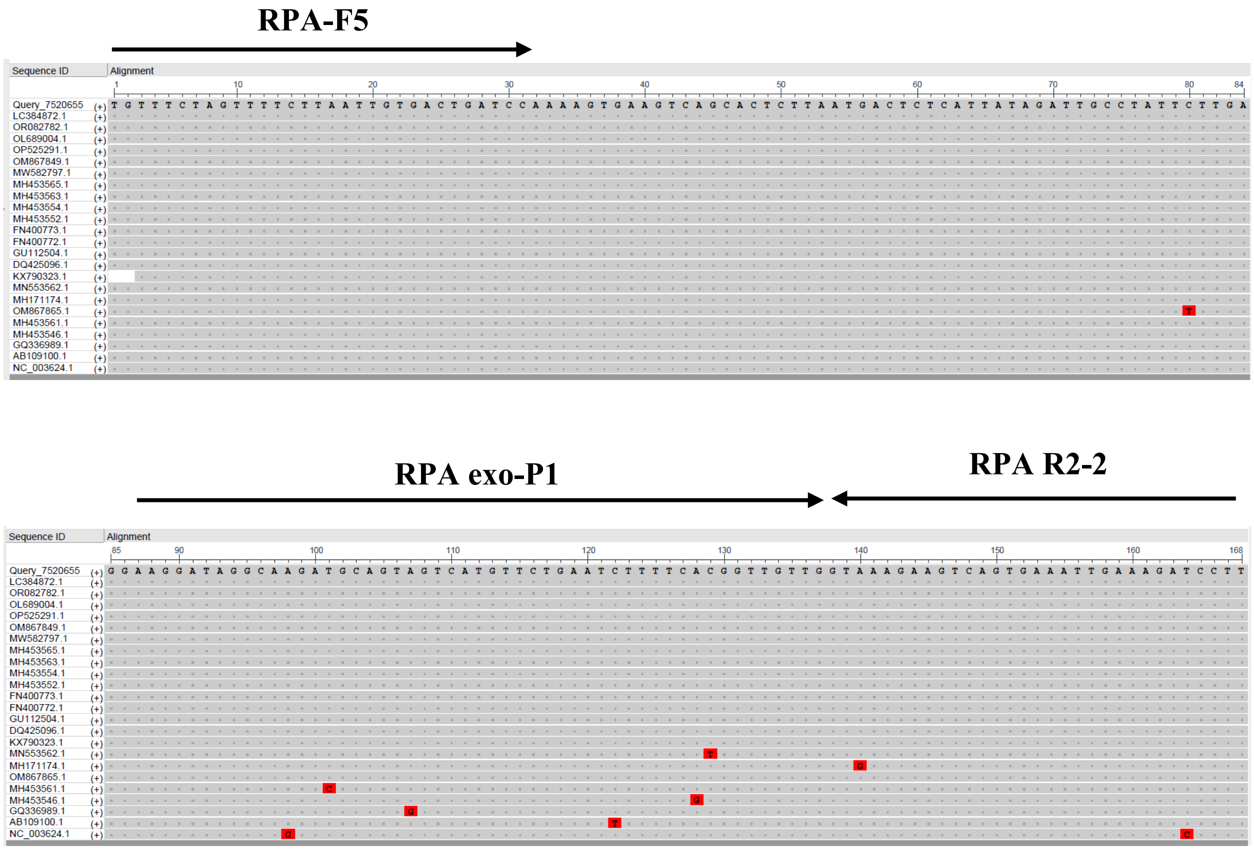

Figure S2. Detection of INSV from crude extracts of thrips prepared in various extraction buffers using RT-RPA.

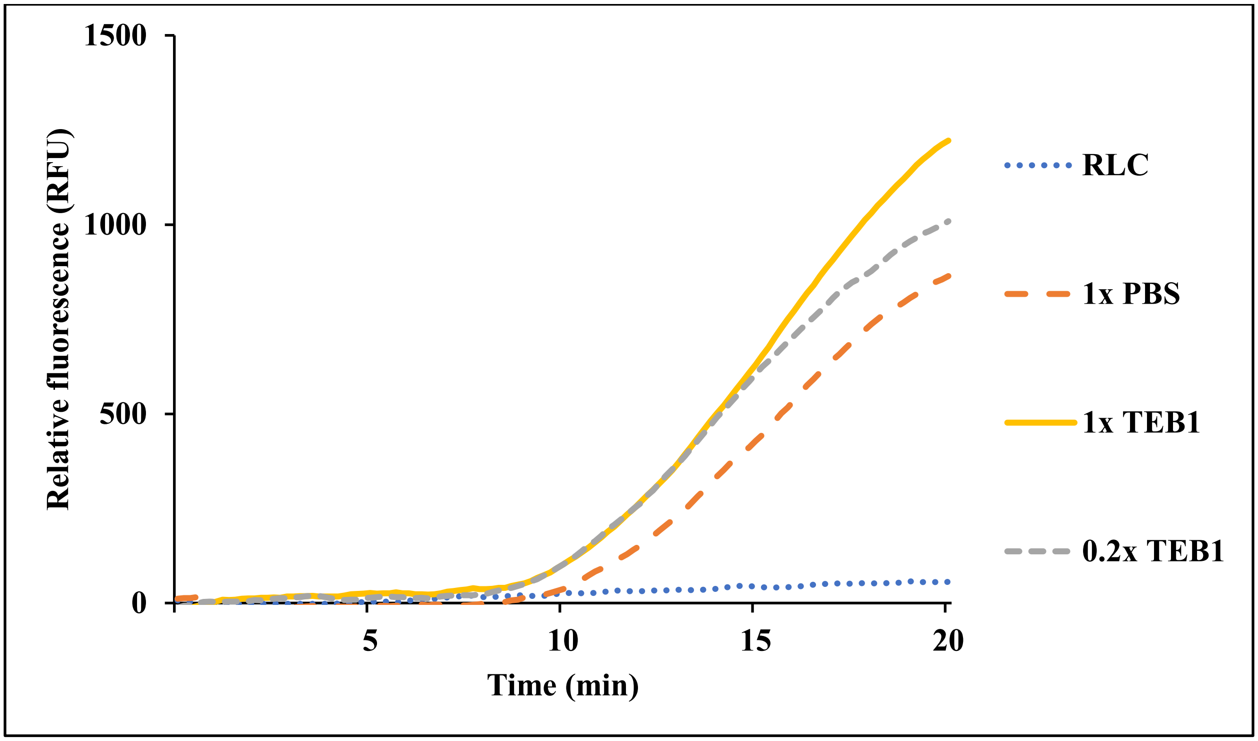

Figure S3. Detection of INSV from *in vitro* transcribed RNA using RT-RPA.

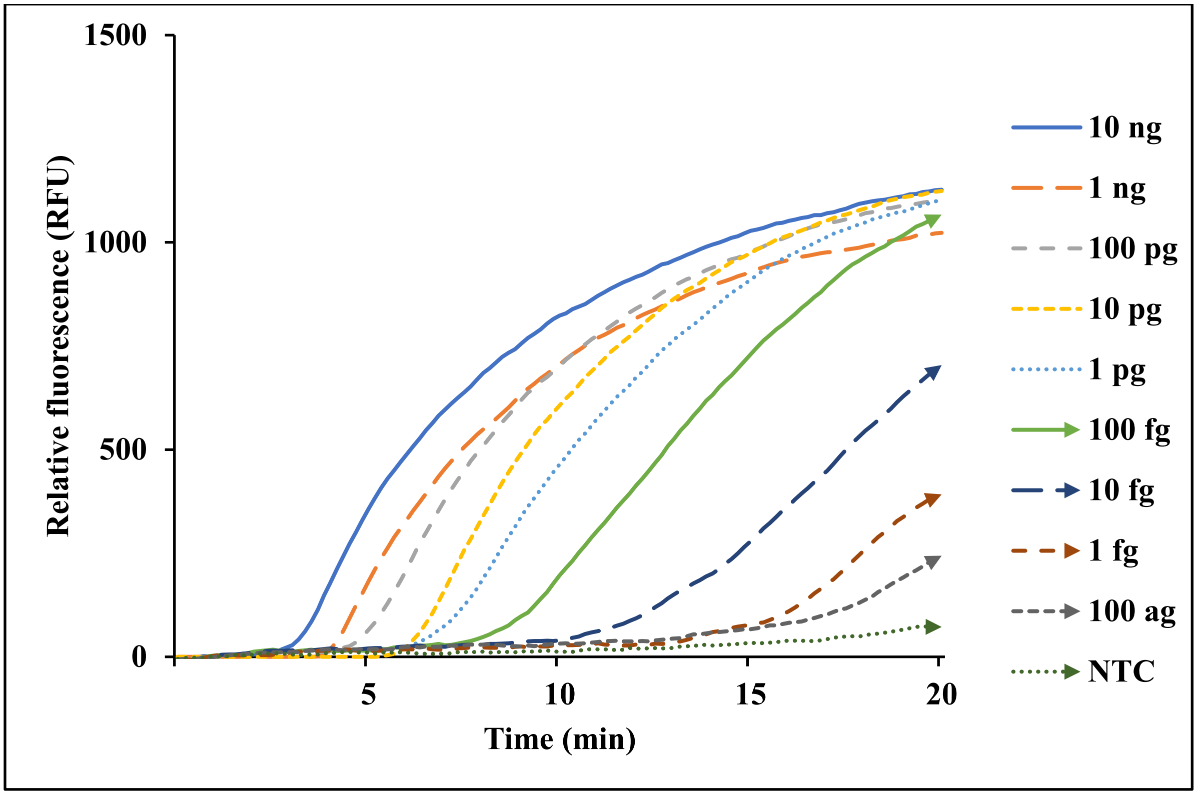

Figure S4. Standard curve of RT-RPA from serial dilutions of the *in vitro* transcribed RNA.

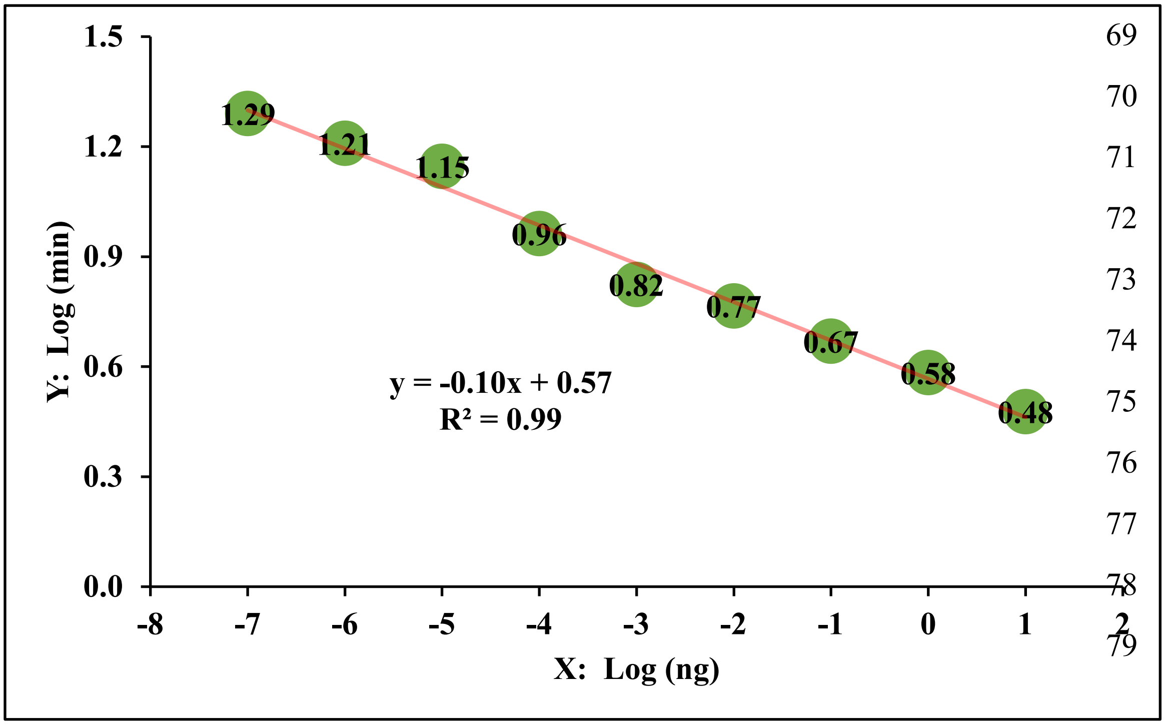

Table S1. List of the primers and probes used for RT-RPA, RT-qPCR, and RT-PCR assays in the study.

Assay	Name	Nucleotide sequence (5′ to 3′)	Location
RT-RPA	INSV RPA-F5	GTTTCTAGTTTTCTTAATTGTGACTGATCCA	Nt #2591-2622
	INSV RPA-R2-2	AAGGATCTTTCAATTTCACTGACTTCTTTA	Nt #2729-2758
	INSV RPA exo-P1	AAGGATAGGCAAGATGCAGTAGTCATGT/iFluorT//idSp/T/ZEN/GAATCTTTTCACGGTTGTTG/3IABkFQ	Nt #2677-2727
RT-qPCR	INSV qPCR-F	GAGGGTGACTTGGAAGGCTAT	Nt #2456-2476
	INSV qPCR-R	TGATGCAATGGTGAGAGTTCA	Nt #2527-2547
	INSV qPCR-P1	FAM-AGCTTCAGTCAGAGCAGATCCATTGTC-BHQ1	Nt #2481-2507
RT-PCR	INSV PCR-F	ACACCCAAGACACAGGATTT	Nt #2401-2420
	INSV PCR-R	CCAAATACTACTTTAACCGCAAGT	Nt #2901-2924

Table S2. List of 24 INSV strains and isolates used for the sequence alignments in this study.

Sequence ID	Strain/Isolate	Country of origin	Host plant species
NC_003624	Strain NL-07	The Netherlands	*Impatiens* sp.
LC384872	Pepe	South Korea	*Peperomia obtusifolia*
OR082782	DSMZ PV-1189	Germany-Bayern	*Ocimum basilicum*
OL689004	ND	China	*Fatsia japonica*
OP525291	DSMZ PV-0485	Unknown	*Gloxinia* sp.
OM867849	DSMZ PV-0281	Germany	*Anemone coronaria*
MW582797	DSMZ PV-0280	USA-East Coast	*Hippeastrum* sp.
MH453567	Lisianthus	USA-CA	*Eustoma* sp.
MH453565	Impatiens	USA-WA	*Impatiens* sp.
MH453563	Green trick	USA-CA	*Dianthus* sp.
MH453554	16-153	USA-NY	*Hosta* sp.
MH453552	16-149	USA-NY	*Digitalis* sp.
FN400773	YSMi-SH	China-Yunnan	*Dendrobium* sp.
FN400772	YSMi-WX	China-Yunnan	*Oncidium* sp.
GU112504	HDL	China	*Hymenocallis littoralis*
DQ425096	Tomato	Italy-Apulia	Tomato
KX790323	Basil-A	USA-WA	*Ocimum basilicum*
MN553562	L7075Gr	Greece	Lettuce
MH171174	UP01	USA-PA	*Impatiens sp.*
OM867865	DSMZ PV-1097	Taiwan	*Nicotiana benthamiana* (lab)
MH453561	Begonia	USA-WA	*Begonia* x *tuberhybrida*
MH453546	14-283	USA-NY	*Hoya wayetii*
GQ336989	Phalaenopsis	China	*Phalaenopsis amabilis*
AB109100	Strain J	Japan	*Verbena* sp.

Table S3. Detection of INSV from plant samples by both RT-qPCR and DAS-ELISA.

SAMPLE	INSV ELISA (abs @ 405nm*)	INSV RT-qPCR (Cq Value**)
Blank – Negative Control	0.091	> 35
Positive Control	3.749	16.13
Lettuce 1	0.576	29.44
Lettuce 2	3.776	14.23
Lettuce 3	3.775	14.12
Lettuce 4	3.815	14.36
*Absorbance readings > 2.5X negative control are considered positive		
**Cq values > 35 = Negative. Assay: 97.3% PCR efficiency; R^2 = 1.000.		
